# The Milan Score is an Effective Manometric Tool to Predict Gastroesophageal Reflux in Patients With Laryngopharyngeal Symptoms

**DOI:** 10.1111/nmo.70015

**Published:** 2025-05-02

**Authors:** Stefano Siboni, Marco Sozzi, Pierfrancesco Visaggi, Ivan Kristo, Nicola De Bortoli, Salvatore Tolone, Elisa Marabotto, Daniele Bernardi, Sebastian F. Schoppmann, Roberto Penagini, Benjamin Rogers, Anthony Hobson, Jordan Haworth, Brian Louie, Yeong Yeh Lee, Vincent Tee, Takahiro Masuda, Dimitrios Theodorou, Tania Triantafyllou, Benedetta Barcella, Lorenzo Cusmai, Michele Puricelli, Marina Coletta, Vito Annese, Edoardo Vincenzo Savarino, Emanuele Luigi Giuseppe Asti, C. Prakash Gyawali

**Affiliations:** ^1^ Division of General and Emergency Surgery, IRCCS Policlinico San Donato University of Milan San Donato Milanese Italy; ^2^ Division of Gastroenterology University of Pisa Pisa Italy; ^3^ Upper‐GI‐Service Medizinische Universität Wien Austria; ^4^ Division of General, Mini‐Invasive and Bariatric Surgery University of Naples Naples Italy; ^5^ Gastroenterology Unit IRCCS Policlinico San Martino Genoa Italy; ^6^ Gastroenterology and Endoscopy Unit Fondazione IRCCS Ca' Granda Ospedale Maggiore Policlinico Milan Italy; ^7^ Division of Gastroenterology Washington University School of Medicine St. Louis Missouri USA; ^8^ The Functional Gut Clinic London UK; ^9^ Division of Thoracic Surgery, Swedish Medical Center Digestive Health Institute Seattle WA USA; ^10^ School of Medical Sciences and GI Function and Motility Unit Universiti Sains Malaysia Kota Bharu Malaysia; ^11^ Department of Surgery Jikei University School of Medicine Tokyo Japan; ^12^ Foregut Surgery Unit University of Athens School of Medicine Athens Greece; ^13^ Division of Gastroenterology, IRCCS Policlinico San Donato University Vita‐Salute S. Rafael Milan Italy; ^14^ Division of Gastroenterology, Department of Surgical, Oncological and Gastroenterological Sciences University of Padua Padua Italy

**Keywords:** gastroesophageal reflux disease, high‐resolution manometry, laryngopharyngeal symptoms

## Abstract

**Introduction:**

According to Lyon 2.0, laryngopharyngeal symptoms (LPS) should undergo upfront pathophysiologic tests. The novel Milan score integrates esophagogastric junction (EGJ) morphology, ineffective esophageal motility, EGJ‐contractile integral (EGJ‐CI), and straight leg raise (SLR) response. It has been demonstrated to predict abnormal AET. The aim of this study was to assess the value of the Milan score in predicting GERD in these patients.

**Methods:**

We prospectively enrolled patients with suspected GERD who underwent HRM and MII pH from 12 referral centers. Patients with isolated LPS (reflux symptom index > 13) were compared with typical GERD symptoms (GERD‐HRQL ≥ 10). A Milan score > 137 was considered positive. The effectiveness of the Milan score in the identification of patients with pathologic GERD was assessed.

**Results:**

Of 570 patients (49% females, median age 49 years, BMI 24 kg/m^2^), isolated LPS was found in 30 patients and isolated typical symptoms in 154. An AET > 6% was found in 23% of the LPS group and 43% of the typical symptom group (*p* = 0.034). The Milan score was higher in the typical symptoms group, with higher scores for EGJ‐CI (*p* = 0.039) and SLR response (*p* = 0.038) components. The likelihood of concordance of the Milan score and AET on reflux monitoring was similar (83.3% vs. 84.4%, *p* = 0.532).

**Conclusions:**

Patients with isolated LPS demonstrated a lower likelihood of EGJ disruption, pathologic GERD, and abnormal Milan score. The Milan score performed similarly well in the identification of GERD in both LPS and typical symptoms and could therefore be used as an upfront test in LPS patients.

## Graphical abstract

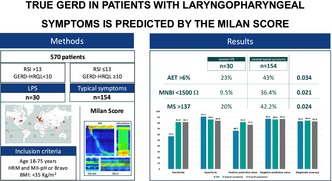

**Summary**

- Patients with isolated LPS demonstrated a lower likelihood of EGJ disruption, pathologic GERD, and abnormal Milan score.
- The Milan score performed similarly well in the identification of GERD in both LPS and typical symptoms.
- The Milan score could therefore be used as an upfront test in LPS patients.

## 1 Introduction

Laryngopharyngeal symptoms (LPS) consist of complaints such as cough, asthma, or hoarseness, which could result from backflow of gastric contents to the upper aerodigestive tract in some individuals [1]. The reporting of LPS is increasing in the general population, with 69% of voice clinic consultations in 2002, compared to 10% in the 1990s [2], and a higher impact on social and work performance compared to gastroesophageal reflux disease (GERD) with typical symptoms [2]. The identification of true GERD‐related LPS is particularly challenging due to the non‐specific clinical presentations included within the LPS spectrum. Although not universally accepted, the recent Lyon 2.0 consensus states that when nonesophageal causes for LPS are ruled out, patients should undergo upfront esophageal physiologic tests to evaluate for conclusive evidence of reflux prior to medical or surgical management [3, 4]. Of note, this approach is gaining popularity in order to limit overprescription of empirical PPI therapy in clinical practice [5].

The diagnosis of actionable GERD is confirmed in patients with Los Angeles Grade B, C, or D esophagitis on endoscopy, and off‐PPI acid exposure time (AET) > 6% on multichannel intraluminal impedance‐pH monitoring (MII‐pH) or at least 2 days with AET > 6% on wireless pH monitoring [3]. Alternative tests for the investigation of patients with LPS, including evaluation of salivary pepsin, laryngoscopy, and pharyngeal pH monitoring using Restech technology, have not proved to be clinically discriminative, without diagnostic power to support a definitive diagnosis [6, 7, 8]. Although ambulatory reflux monitoring has demonstrable value, catheter‐based pH‐impedance studies are time‐consuming and can be troublesome for LPS patients with throat clearing and cough, while prolonged wireless pH monitoring adds additional cost [9]. The medical community is therefore striving to find a simple and reliable tool that predicts true GERD in patients with LPS.

Thus far, high‐resolution manometry (HRM) has only a secondary role in the diagnostic work‐up of GERD, providing adjunctive evidence when metrics from ambulatory reflux monitoring are inconclusive. Recently, the novel Milan score, generated from HRM evaluation of esophagogastric junction (EGJ) morphology, ineffective esophageal motility (IEM), EGJ‐contractile integral (EGJ‐CI), and straight leg raise (SLR) response, has been demonstrated to predict abnormal AET, stratifying the risk of GERD [10]. If these findings are replicated in LPS, the upfront use of the Milan score could make HRM a useful tool to identify LPS patients with low risk of GERD, who can then be channeled to alternate therapeutic pathways, reserving reflux monitoring, and acid‐suppressive therapies for patients with high risk of GERD [5].

We hypothesized that the Milan score would effectively segregate LPS patients with true GERD from those without risk for GERD. Therefore, the aim of this exploratory study was to describe HRM characteristics in patients with LPS and to assess the value of the Milan score in predicting GERD in these patients.

## 2 Material and Methods

**FIGURE 1:**
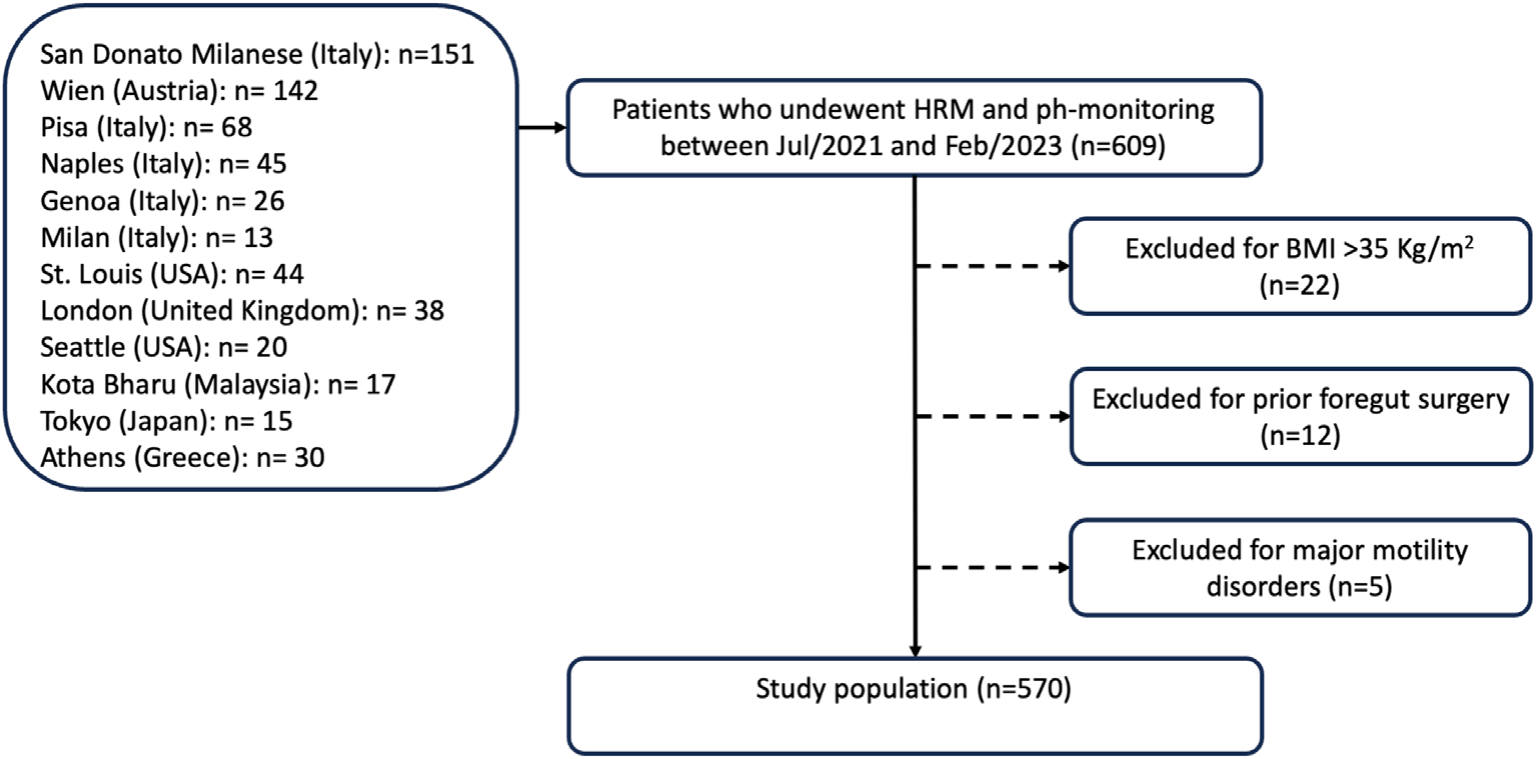
Study flow chart.

All patients with suspected GERD between ages 18 and 75 years who underwent HRM and MII‐pH or wireless pH study within 2 weeks of each other were prospectively enrolled from 12 referral centers worldwide between July 2021 and February 2023. Reasons for reflux monitoring included assessment of candidacy for antireflux surgery, need for long‐term medical treatment, or refractoriness to PPI therapy. Exclusion criteria consisted of body mass index (BMI) > 35 kg/m^2^, pregnancy, eosinophilic esophagitis, connective tissue disorders, achalasia, prior foregut surgery, and studies with artifacts precluding interpretation. The study flow chart is presented in Figure 1.

A thorough clinical evaluation was performed in conjunction with esophageal physiologic testing. Demographic and clinical characteristics including age, BMI, primary and secondary symptoms, PPI use and response, and upper endoscopy findings (hiatal hernia, esophagitis, and Barrett esophagus) were abstracted from each patient's medical record. Esophagitis was classified according to the Los Angeles classification as follows: Grade A as one or more mucosal erosions no longer than 5 mm, Grade B as erosions longer than 5 mm, Grade C as erosions extending < 75% of the circumference, and Grade D as erosions involving ≥ 75% of the esophageal circumference. Barrett esophagus was defined as salmon‐colored mucosa in the distal esophagus with histologically confirmed intestinal metaplasia. Self‐report validated questionnaires were administered to categorize the symptom pattern, including GERDQ [11], GERD health‐related quality of life (GERD‐HRQL) [12], and reflux symptom index (RSI) [13].

The study protocol was approved by local institutional review boards (IRB) at each collaborating center. An online secure de‐identified database was utilized for data upload and data sharing. The study was conducted in accordance with the Helsinki Declaration. All authors had access to the study data; each author reviewed and approved the final manuscript.

### 2.1 High‐Resolution Esophageal Manometry

High‐resolution manometry was performed using a solid‐state catheter with 36 circumferentially arranged sensors at 1‐cm intervals after an overnight fast. Each test was conducted in accordance with the Chicago Classification 4.0 (CCv4.0) standard protocol [14], with multiple rapid swallows (MRS) and straight leg raise (SLR) maneuvers as required adjunctive provocative tests.

Ten swallows of 5‐mL room‐temperature water were performed in the primary position (upright or recumbent) according to each center's preference, followed by five swallows in the secondary position. Based on distal contractile integral (DCI) values, each swallow was classified as intact, weak, or failed (≥ 450 mmHg•cm•s, 100–450 mmHg•cm•s, and ≤ 100 mmHg•cm•s, respectively). Patients with > 70% weak swallows or ≥ 50% failed swallows were categorized as IEM. EGJ morphology was classified as Type 1 if the LES and the crura diaphragm (CD) high‐pressure zones were superimposed, Type 2 if the LES‐CD separation was < 3 cm, and Type 3 if it was ≥ 3 cm. All the baseline characteristics, such as LES basal and resting pressure, LES total and intra‐abdominal length, and EGJ‐contractile integral (EGJ‐CI) were recorded and collected. EGJ‐CI was calculated during the reference period using the DCI software tool over the EGJ and corrected for respiration and gastric pressure [15]. Basal upper esophageal sphincter contractile integral (UES‐CI) was calculated using the DCI box over the UES for three respiratory cycles, divided by the time [16].

MRS consisted of five swallows of 2‐mL room‐temperature water administered at < 3 s intervals. Contractile reserve was present when the ratio between MRS DCI and mean single swallow DCI was > 1. The SLR maneuver was performed by having the patients raise one or both legs to obtain an increase of intra‐abdominal pressure of 50% or more compared to the resting position. An increase of esophageal peak pressure recorded 5 cm above the proximal margin of the LES of at least 11 mmHg during SLR was considered abnormal [17].

### 2.2 Milan Score

**FIGURE 2:**
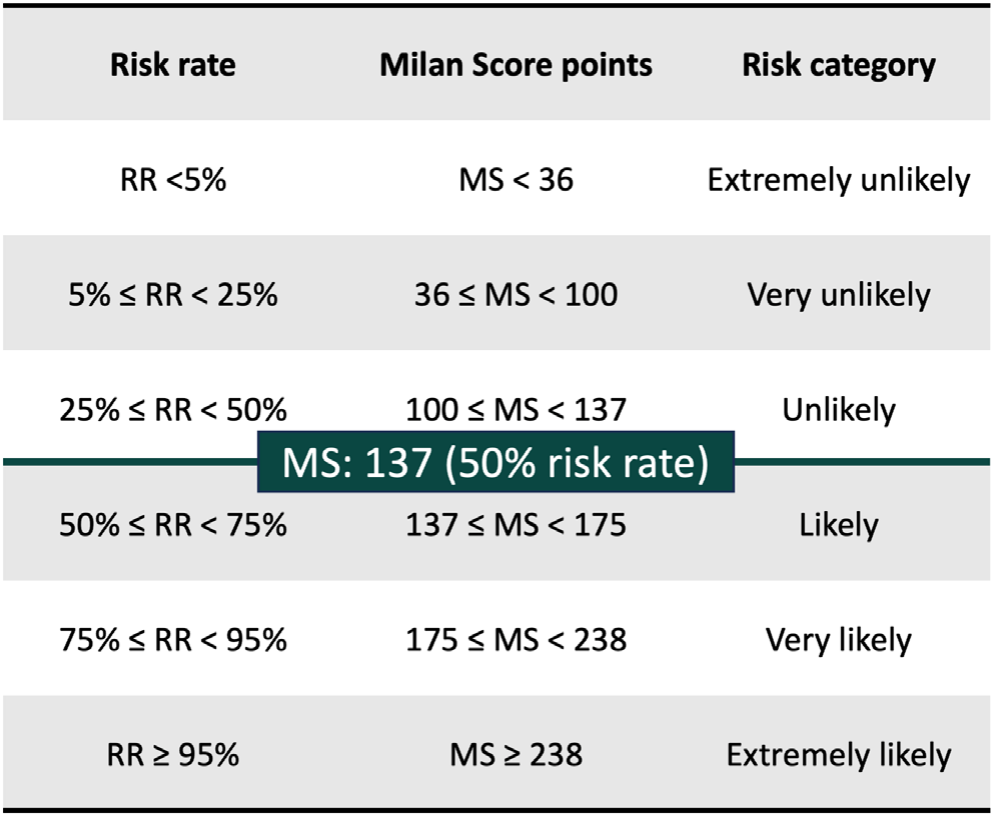
Examples of the use of the Milan score (www.milanscore.com).

The Milan score was developed to predict pathologic GERD and to stratify disease severity using four variables collected during HRM (IEM presence, EGJ‐CI value, EGJ morphology, and SLR response). A score is assigned to each variable, calculated using an online and mobile app software (www.milanscore.com), based on a previously validated statistical model [10]. The sum of the four scores provides the final Milan score, where a higher value corresponds to higher degrees of disruption of the antireflux barrier and reliably predicts AET > 6%. The Milan score, therefore, also segregates mild from severe reflux, since it describes the degree of EGJ barrier disruption as well as the potential for abnormal esophageal clearance due to inefficient motility. The distribution of the risk rate for GERD according to the Milan score identifies six classes of risk, from extremely unlikely to extremely likely (Figure 2).

### 2.3 Esophageal pH and pH‐Impedance Study

Catheter‐based reflux monitoring and wireless 48 or 96 h pH studies were both allowed according to each center's preference [18]. All pH studies were performed after at least 14 days of PPI withdrawal. During this time, patients were allowed to take antacids and alginate‐based compounds as rescue medication [19]. The wireless pH capsule (BRAVO) was positioned 6 cm above the squamocolumnar junction during endoscopy. The MII‐pH studies were performed using catheters with eight impedance and one or two pH electrodes, calibrated using buffer solutions at pH 4.0 and 7.0, and then inserted transnasally. The pH electrode was positioned 5 cm above the proximal margin of the LES. The patient was instructed to avoid acidic food and drinks during the recording time and to document the duration of meals, time spent in the recumbent position, and symptoms.

Total, upright, and recumbent AET, the number of acid, weakly acid, and weakly alkaline reflux episodes (with MII‐pH studies), DeMeester score, symptom index (SI), and symptom association probability (SAP) were recorded, as previously described [20]. Reflux–symptom association was defined as SI > 50% and SAP > 95%. As indicated by Lyon 2.0, MNBI was collected from pH‐impedance studies. The postreflux swallow‐induced peristaltic wave (PSPW) index was also collected [21]. All the physicians evaluating the pH tracings were blinded to the Milan score results.

### 2.4 Data Collection and Statistical Analysis

All participating centers uploaded de‐identified data on the secure online Research Electronic Data Capture (RedCap) platform. Categorical variables are reported as count and percentage, while numerical variables are reported as median and interquartile range (IQR). The normality of continuous variables was assessed using the Shapiro–Wilk test. The Chi‐square test or Fisher test was used as appropriate for categorical values, while continuous values were compared using a nonparametric Kruskal–Wallis test. The outcome variable was pathologic reflux defined as AET > 6% on MII‐pH or ≥ 2 days with AET > 6% on wireless monitoring. In the case of borderline reflux (AET between 4% and 6%) adjunctive MII‐pH metrics were used. MNBI < 1500 Ω and total reflux episodes > 80 were considered pathologic.

Among the study population, patients with isolated LPS were defined as RSI > 13 and GERD‐HRQL < 10, while patients reporting isolated typical GERD symptoms had GERD‐HRQL ≥ 10 and RSI ≤ 13. In order to compute the Milan score, manometric characteristics including EGJ morphology, IEM, EGJ‐CI, and SLR response were reviewed and compared. A Milan score of 137 or higher, corresponding to a 50% risk of AET > 6%, was considered positive. The effectiveness of the Milan score in the identification of patients with pathologic GERD was assessed in the two patient groups as well as in the group of patients with both symptom patterns (RSI > 13 and GERD‐HRQL ≥ 10). Sensitivity and specificity analysis were performed comparing a positive Milan score with pathologic GERD on reflux monitoring studies in each group as well as in the overall study population. A two‐tailed *p* < 0.05 was considered significant for all statistical tests. Statistical analyses were performed using SAS version 9.4 (SAS Institute, Cary, North Carolina).

## 3 Results

Of 570 total study patients, isolated LPS was found in 30 patients, and isolated typical GERD symptoms in 154 patients. The remainder, 386 patients, presented either with both symptom patterns (138 patients) or with mild symptoms not reaching the criteria to fit in either study group (248 patients). Patients with LPS (*n* = 30) were older (54 years vs. 48 years, *p* = 0.024), but gender distribution, BMI, duration of symptoms, and PPI use were similar between patients with LPS and typical reflux symptoms (Table 1). Of note, PPI response was significantly lower in the LPS group compared to patients with typical symptoms (*p* = 0.009).

**TABLE 1:** Clinical characteristics of the study populations.

	Overall (*n* = 184)	LPS (*n* = 30)	Typical symptoms (*n* = 154)	*p*
Age, (years)	49 [24]	54 [22]	48 [24]	0.024
Female, *n* (%)	89 (49.2)	16 (55.2)	73 (48)	0.546
BMI, (Kg/m^2^)	24.2 [4.9]	24.7 [5.2]	24.2 [4.8]	0.424
Symptoms duration, (months)	30 [48]	28 [42]	32 [49]	0.381
PPI use, *n* (%)	161 (91.0)	25 (89.3)	136 (91.3)	0.722
PPI response				0.009
No benefit, *n* (%)	46 (28.6)	13 (52.0)	33 (24.3)	
Partial benefit, *n* (%)	71 (44.1)	10 (40.0)	61 (44.9)	
Full benefit, *n* (%)	44 (27.3)	2 (8.0)	42 (30.9)	

*Note:* Continuous values are expressed as median [IQR].

Abbreviations: BMI, body mass index; LPS, laryngo‐pharyngeal symptoms; PPI, proton‐pump inhibitors.

Variables from endoscopy, HRM, and pH‐monitoring analysis are shown in Table 2. The likelihood of an endoscopic hiatus hernia was lower in the isolated LPS group (*p* = 0.034). Additionally, patients in the isolated LPS group had lower median UES basal pressure (*p* = 0.025), lower median UES‐CI (*p* = 0.024), lower rates of positive SLR response (23.3% vs. 42.2%, *p* = 0.039), and a higher median EGJ‐CI (49.5 vs. 31.8 mmHg*cm, *p* = 0.020) compared to patients with typical symptoms.

**TABLE 2:** Endoscopy, HRM, and reflux monitoring characteristics of the study populations.

	Overall (*n* = 184)	LPS (*n* = 30)	Typical symptoms (*n* = 154)	*p*
ENDOSCOPY				
Hiatal hernia, *n* (%)	111 (64.9)	14 (46.7)	97 (68.8)	0.034
Esophagitis, *n* (%)	50 (29.2)	5 (17.9)	45 (31.5)	0.177
Grade A, *n* (%)	21 (42.0)	2 (40.0)	19 (42.2)	0.051
Grade B, *n* (%)	24 (48.0)	1 (20.0)	23 (51.1)	
Grade C, *n* (%)	2 (4.0)	0 (0.0)	2 (4.4)	
Grade D, *n* (%)	3 (6.0)	2 (40.0)	1 (2.2)	
Barrett esophagus	4 (2.4)	2 (6.9)	2 (1.4)	0.137
HRM				
EGJ type				0.605
1, *n* (%)	96 (52.2)	18 (60.0)	78 (50.6)	
2, *n* (%)	60 (32.6)	9 (30.0)	51 (33.1)	
3, *n* (%)	28 (15.2)	3 (10.0)	25 (16.2)	
Hiatal hernia, *n* (%)	71 (39.7)	11 (36.7)	60 (40.3)	0.839
Hiatal hernia size, (cm)	1.6 [2.0]	1.5 [1.4]	1.6 [2.5]	0.093
LES total length, (cm)	2.1 [0.7]	2.0 [0.7]	2.1 [0.7]	0.437
LES intrabdominal length, (cm)	0.3 [1.0]	0.8 [1.4]	0.2 [0.9]	0.074
EGJ‐ CI, (mmHg*cm)	33.2 [31.5]	49.5 [45.7]	31.8 [29.9]	0.020
Patients with IEM, n, (%)	38 (20.7)	4 (13.3)	34 (22.1)	0.334
UES basal pressure, (mmHg)	87.2 [57.4]	63.1 [46.4]	89.6 [61.5]	0.025
UES residual pressure, (mmHg)	−3.4 [9.5]	0.5 [11.1]	−4.9 [9.3]	0.451
UES‐CI, (mmHg*cm)	97.0 [88.9]	78.1 [33.7]	118.4 [81.4]	0.024
Positive SLR, *n* (%)	72 (39.1)	7 (23.3)	65 (42.2)	0.039
Milan Score Components				
Score EGJ type	14.9 ± 17.2	11.9 ± 16.1	15.6 ± 17.4	0.284
Score IEM	5.5 ± 10.8	3.6 ± 9.3	5.9 ± 11.2	0.227
Score EGJ‐CI	53.2 ± 8.5	49.9 ± 12.1	54.8 ± 6.7	0.039
Score SLR	42.3 ± 62.3	23.3 ± 43.1	42.2 ± 49.5	0.038
Score total	115.9 ± 62.3	88.7 ± 51.5	118.6 ± 65.6	0.008
Risk rate (RR), (%)	40.4 ± 32.4	25.8 ± 27.5	41.6 ± 34.8	0.009
Patients with RR > 50%, *n* (%)	71 (38.6)	6 (20)	65 (42.2)	0.024
pH‐impedance monitoring				
Acid exposure time, (%)	4.1 [7.0]	2.1 [3.3]	4.3 [7.8]	< 0.001
Acid exposure time, upright, (%)	5.4 [9.4]	2.8 [7.4]	5.7 [9.5]	0.034
Acid exposure time, recumbent, (%)	0.9 [11.8]	0.2 [1.7]	1.1 [6.0]	< 0.001
AET > 6%, *n*, (%)	73 (39.7)	7 (23.3)	66 (42.9)	0.046
DeMeester score	16.7 [24.9]	9.2 [18.4]	17.9 [28.5]	< 0.001
Total reflux episodes, (n)	37 [41]	32 [33]	39 [43]	0.366
Acid reflux episodes, (n)	25.5 [30.0]	17.0 [20.5]	27.0 [34.0]	0.017
Proximal reflux, *n* (%)	62/77 (80.5)	11/13 [84.6]	51/64 (79.9)	0.512
MNBI, (Ω)	2257 [1196]	2400 [1332]	1980 [2293]	0.152
MNBI < 1500, *n* (%)	46 (32.4)	2 (9.5)	44 (36.4)	0.021
PSPW index, (%)	50.0 [37.5]	65.0 [41.3]	49.0 [38.0]	0.086
Reflux–symptom association, (%)	34 (22.5)	6 (26.1)	28 (21.9)	0.786

*Note:* Continuous values are expressed as median [IQR].

Abbreviations: AET, acid exposure time; EGJ, esophago‐gastric junction; EGJ‐CI, esophago‐gastric junction contractile integral; IEM, ineffective esophageal motility; LES, lower esophageal sphincter; LPS, laryngo‐pharyngeal symptoms; MNBI, mean nocturnal baseline impedance; PSPW, postreflux swallow‐induced peristaltic wave; UES, upper‐esophageal sphincter; UES‐CI, upper esophageal sphincter contractile integral.

A similar rate of patients with IEM was found in the two groups (*p* = 0.334), and no major motility disorders were found.

MII‐pH analysis showed higher total, upright, and recumbent AET, higher DeMeester score, higher median number of acid reflux episodes (17 vs. 27, *p* = 0.017), and a higher number of patients with MNBI < 1500 Ω in the isolated typical symptoms group compared to LPS patients. Data on proximal reflux were recorded in 77 patients (42%), and no significant differences were found between the groups (84.6% in LPS vs. 79.9% in typical symptom group, *p* = 0.512).

When the individual components of the Milan score were separately analyzed, a significantly higher EGJ‐CI and SLR response was found in the typical symptom group, with a higher median Milan score (88.7 vs. 118.6, *p* = 0.008) and, consequently, a higher risk of pathologic reflux (25.8% vs. 41.6%, *p* = 0.009). Furthermore, patients with a risk rate of reflux of ≥ 50% (Milan score ≥ 137) were significantly higher among the typical symptom group (42.2% vs. 20%, *p* = 0.024) (Table 2). When the Milan score was analyzed according to risk category, we found a higher rate of LPS patients in the class “very unlikely” (73.3% vs. 50.6%, *p* = 0.027) and a higher rate of typical symptom patients in the class “very likely” (31.8% vs. 13.3%, *p* = 0.029) (Table 3).

**TABLE 3:** Milan score components, total points, and risk rate.

Milan score category	Total (*n* = 184)	LPS (*n* = 30)	Typical symptoms (*n* = 154)	*p*
Extremely unlikely, *n* (%)	2 (1.1)	0 (0.0)	2 (1.3)	1.000
Very unlikely, *n* (%)	100 (54.3)	22 (73.3)	78 (50.6)	0.027
Unlikely, *n* (%)	11 (6)	2 (6.7)	9 (5.8)	0.563
Likely, *n* (%)	17 (9.2)	2 (6.7)	15 (9.7)	0.452
Very likely, *n* (%)	53 (28.8)	4 (13.3)	49 (31.8)	0.029
Extremely likely, *n* (%)	1 (0.5)	0 (0.0)	1 (0.6)	1.000

Abbreviation: LPS, laryngo‐pharyngeal symptoms.

**FIGURE 3:**
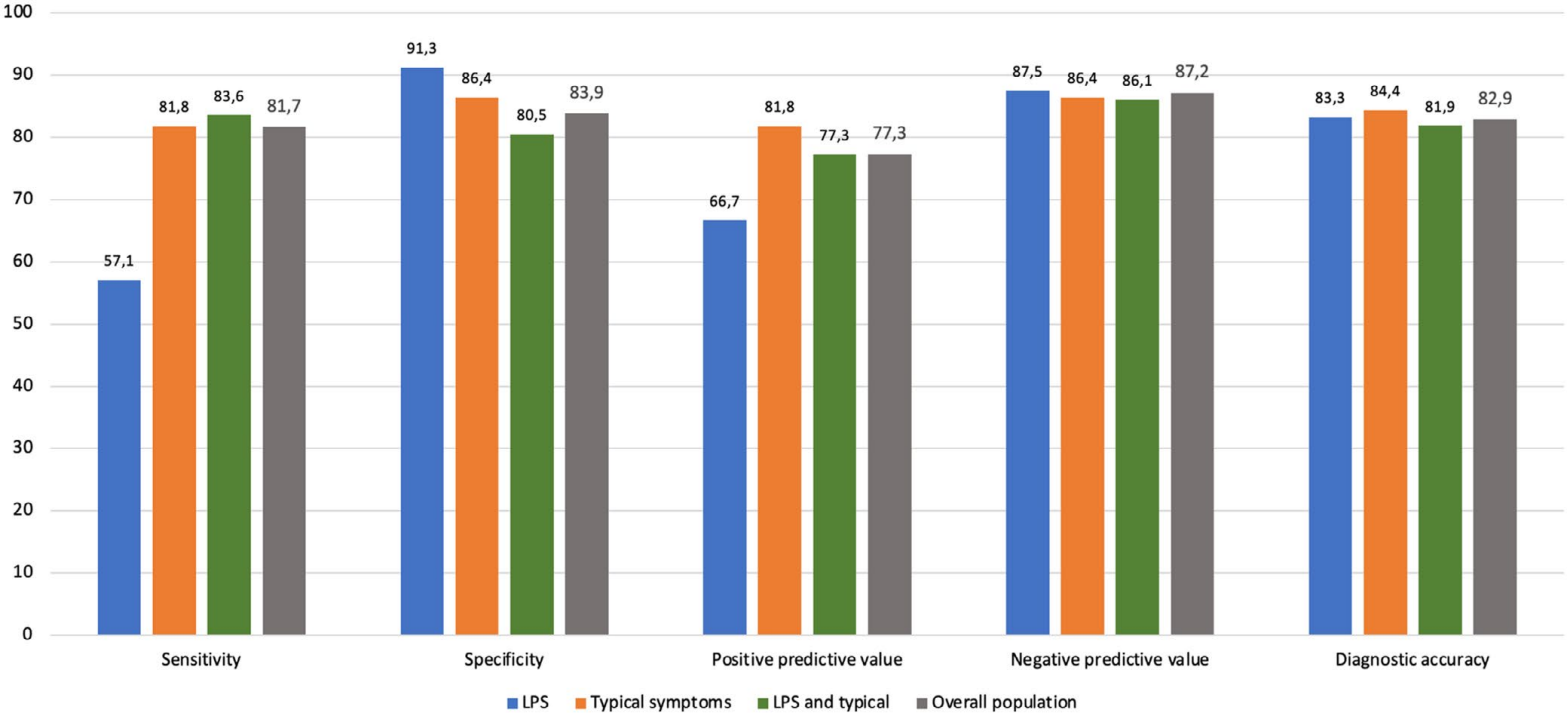
Comparison of performance metrics of Milan score for the diagnosis of GERD in LPS versus typical symptoms.

Among patients with LPS, the Milan score had a lower sensitivity (57.1%) but higher specificity (91.3%) for the diagnosis of conclusive GERD compared to patients with typical symptoms (sensitivity 81.8% and specificity 86.4%), with similar diagnostic accuracy (83.3% in LPS group and 84.4% in typical symptoms group). In patients with both LPS and typical symptoms, the Milan score showed a sensitivity of 83.6% and a specificity of 80.5%, with similar diagnostic accuracy (81.9%). The negative predictive value of the Milan score in the LPS group was 87.5% (Figure 3). Sensitivity and specificity of complete response to PPI were 16.7% and 94.7% in the LPS group and 45.9% and 81.3% in the typical symptom group, with diagnostic accuracy of 76% and 65.4%, respectively (Figure S1).

## 4 Discussion

In this study evaluating the value of the Milan score in patients with isolated LPS compared to isolated typical GERD symptoms, we demonstrate that the Milan score is useful in the assessment of the risk of pathologic GERD in patients with LPS. The diagnostic accuracy of the Milan score in the LPS group was 83.3%, demonstrating good concordance with ambulatory pH‐impedance monitoring. Even though sensitivity in the LPS group was low (57.1%, likely reflecting lower likelihood of pathologic reflux), the diagnostic accuracy was similar to that seen in the typical symptom group (84.4%). Additionally, the negative predictive value of the Milan score was very high (87.5%), indicating that a Milan score towards the low end of the spectrum may indicate a low likelihood of GERD as a mechanism for LPS. Of note, the diagnostic accuracy of the Milan score in predicting a pathologic reflux monitoring test in the whole population was 83.3%, higher than the PPI test (58.6%) and endoscopy (69.2%).

The fact that the Milan score performed similarly well at identifying true GERD in both groups of patients suggests that the underlying pathophysiological causes of GERD, in particular disruption of the antireflux barrier, are similar in patients with LPS and typical GERD with pathologic AET. Moreover, the favorable diagnostic accuracy in both patient groups demonstrates that the diagnostic performance of the Milan score is not exclusive to a particular symptom pattern.

On the other hand, the pathophysiological mechanisms underlying symptoms are different; in fact, patients with typical symptoms had a more compromised EGJ barrier function (median EGJ‐CI 31.8 vs. 49.5 mmHg*cm) and a higher rate of impaired response to SLR maneuver (42.2% vs. 23.3%). These differences explain the dissimilar distribution of the Milan score classes in the groups (Table 3), reflecting a more disrupted antireflux barrier in the typical symptoms group. Further study of the various EGJ barrier disruption subtypes is needed to understand the significance of EGJ barrier disruption in the development of both typical and atypical symptoms.

Although there have been multiple attempts to evaluate the complex pathophysiology of LPS, a singular explanation remains elusive, and both the reflux theory [22] (direct injury from reflux of acid and pepsin into the larynx) and the reflex theory [23] (vagally mediated symptom response with laryngeal hypervigilance) have merits in different patients. In our study comparing two cohorts of patients with isolated LPS and typical symptoms, the latter group had not only a weaker EGJ but also a higher rate of patients with pathologic MNBI (36.4% vs. 9.5%), suggesting more epithelial injury despite the fact that proximal reflux events were similar between the two groups. However, distal MNBI alone should not be considered a predictor for conclusive GERD in LPS, and there was a significant discrepancy between positive pH‐impedance studies (23.3%) and low MNBI (9.5%). As expected, LPS was associated with a lower prevalence of pathologic acid exposure (23.3%) compared to typical symptoms (42.9%), which is consistent with reports in the literature [9, 24]. Esophageal body hypomotility (IEM) prevalence was also lower in LPS patients in our study (13.3%), lower than that reported by other studies (30.9%) using CC v3.0 definitions [25]. The low prevalence of pathologic acid exposure time, MNBI, and IEM in the LPS group and our proximal episode findings may favor the reflex theory rather than pathologic GERD in most LPS patients. Nevertheless, MNBI, especially differential MNBI between distal and proximal esophagus, continues to be evaluated as a marker for LPS [26], and firm conclusions cannot be made until larger LPS cohorts are studied.

While UES metrics were not available in most patients, UES basal metrics were compromised in a proportion of LPS patients, supporting findings from Benjamin et al. [27]. However, our study was not designed to evaluate UES findings, and a more comprehensive analysis of a larger dataset is needed to provide more robust evidence evaluating UES function in LPS.

An important finding in our study is the high specificity (91.3%) and negative predictive value (87.5%) of the Milan score in LPS patients. Although our population is not naive, as most of them already underwent EGD and/or PPI trials, this result suggests that when HRM is performed in LPS, the Milan score can quantify the integrity of the antireflux barrier, which may allow subsets of LPS patients to not require additional esophageal physiologic testing if clinical suspicion for a compromised barrier and GERD is low. However, since the sensitivity of the Milan score for LPS patients is relatively low (57.1%), this metric should not be used as the only diagnostic test in these patients, but pH monitoring should always be considered. In contrast, the high negative predictive value (87.5%) suggests that a low Milan score would implicate non‐GERD mechanisms in LPS, and the utility of ambulatory reflux monitoring may be low.

In case of same‐day physiologic tests, our findings offer, nevertheless, a benefit, providing adjunctive evidence in case of inconclusive diagnosis or to overcome the day‐to‐day variability issue of 24‐h pH monitoring. Moreover, the Milan score may also help to perform a more precise selection of patients who could benefit from laryngeal recalibration therapy, which has shown promising results in a recent paper [28].

Even though HRM is not well tolerated by every patient, the potential gain in terms of ruling out major motility disorders and avoiding over‐prescription of PPI may justify the burden of the test, particularly since HRM may serve to lower the economic burden of unnecessary treatment and tests in LPS patients [29]. Our findings embrace the philosophy of the Lyon 2.0 consensus, where upfront pathophysiologic tests are indicated in patients with LPS, more to exclude than to diagnose GERD [3]. Given the poor response of these “atypical” symptoms to PPIs and antireflux surgery [30], a definitive diagnosis of pathologic reflux with prolonged wireless pH study or MII‐pH monitoring remains an absolute indication before an escalation of medical therapy or, even more, surgical treatment.

The strengths of our study are the inclusion of patients with precise identification of symptom patterns and comprehensive clinical and pathophysiologic evaluation performed at 12 academic centers around the world. Our study has also some limitations, including the small sample size of LPS patients, limited data on UES and proximal reflux metrics, including reflux episodes and proximal MNBI, and the lack of stratification of the population according to EGJ morphology. Since the patient cohorts were collected from tertiary care centers and the patients are not naive, this may impact the generalizability of our findings to the average general practitioner, general surgeon, and gastroenterologist. Moreover, possible regional and racial differences in the interpretation of the Milan score have not been taken into account, despite the recent findings observed in establishing normal values for impedance‐pH monitoring metrics [31]. Finally, a comparison between patients assessed by wireless pH versus catheter based was not performed given the small number (37 patients) of wireless studies. Nevertheless, we feel our findings help explain the low response of LPS patients to acid‐suppressive therapies since we have demonstrated a low likelihood of abnormal reflux pathophysiology in this patient population. Further studies are needed to integrate clinical scores (i.e., COuGH RefluX score) to HRM and pH‐impedance parameters [32].

## 5 Conclusion

In conclusion, patients with isolated LPS demonstrated a lower likelihood of EGJ disruption, pathologic GERD, and abnormal Milan score compared to patients with typical symptoms. The Milan score performed similarly well in the identification of GERD in both isolated LPS patients and patients with typical reflux symptoms and could therefore be used as an upfront test in LPS patients.

## Author Contributions

**Stefano Siboni:** conception of the study, acquisition of the data, analysis and interpretation of the data, drafting the paper, approving the final manuscript. **Marco Sozzi:** conception of the study, acquisition of the data, drafting the paper, approving the final manuscript. **Pierfrancesco Visaggi:** acquisition of the data, drafting the paper, approving the final manuscript. **Ivan Kristo:** conception of the study, acquisition of the data, drafting the paper, approving the final manuscript. **Nicola De Bortoli:** conception of the study, drafting the paper, approving the final manuscript. **Salvatore Tolone:** acquisition of the data, drafting the paper, approving the final manuscript. **Elisa Marabotto:** acquisition of the data, drafting the paper, approving the final manuscript. **Daniele Bernardi:** conception of the study, acquisition of the data, drafting the paper, approving the final manuscript. **Sebastian F. Schoppmann:** conception of the study, drafting the paper, approving the final manuscript. **Roberto Penagini:** conception of the study, drafting the paper, approving the final manuscript. **Benjamin Rogers:** conception of the study, acquisition of the data, drafting the paper, approving the final manuscript. **Anthony Hobson:** conception of the study, acquisition of the data, drafting the paper, approving the final manuscript. **Jordan Haworth:** acquisition of the data, drafting the paper, approving the final manuscript. **Brian Louie:** acquisition of the data, drafting the paper, approving the final manuscript. **Yeong Yeh Lee:** acquisition of the data, drafting the paper, approving the final manuscript. **Vincent Tee:** acquisition of the data, analysis and interpretation of the data, drafting the paper, approving the final manuscript. **Takahiro Masuda:** acquisition of the data, analysis and interpretation of the data, drafting the paper, approving the final manuscript. **Dimitrios Theodorou:** acquisition of the data, drafting the paper, approving the final manuscript. **Tania Triantafyllou:** acquisition of the data, drafting the paper, approving the final manuscript. **Benedetta Barcella:** acquisition of the data, drafting the paper, approving the final manuscript. **Lorenzo Cusmai:** acquisition of the data, drafting the paper, approving the final manuscript. **Michele Puricelli:** acquisition of the data, drafting the paper, approving the final manuscript. **Marina Coletta:** acquisition of the data, drafting the paper, approving the final manuscript. **Vito Annese:** acquisition of the data, drafting the paper, approving the final manuscript. **Edoardo Vincenzo Savarino:** conception of the study, analysis and interpretation of the data, drafting the paper, approving the final manuscript. **Emanuele Luigi Giuseppe Asti:** conception of the study, drafting the paper, approving the final manuscript. **C. Prakash Gyawali:** conception of the study, analysis and interpretation of the data, drafting the paper, approving the final manuscript.

## Conflicts of Interest

The authors declare no conflicts of interest.

## Supporting information


**Figure S1.** Comparison of performance metrics of PPI response for the diagnosis of GERD in LPS versus typical symptoms.

## Data Availability

The data that support the findings of this study are available on request from the corresponding author. The data are not publicly available due to privacy or ethical restrictions.
