# Supplementary figures and images for: The Milan Score is an Effective Manometric Tool to Predict Gastroesophageal Reflux in Patients With Laryngopharyngeal Symptoms
Source: Neurogastroenterol Motil. 2025 May 2;38:e70015. doi: 10.1111/nmo.70015 (PMC13121873; doi:10.1111/nmo.70015)

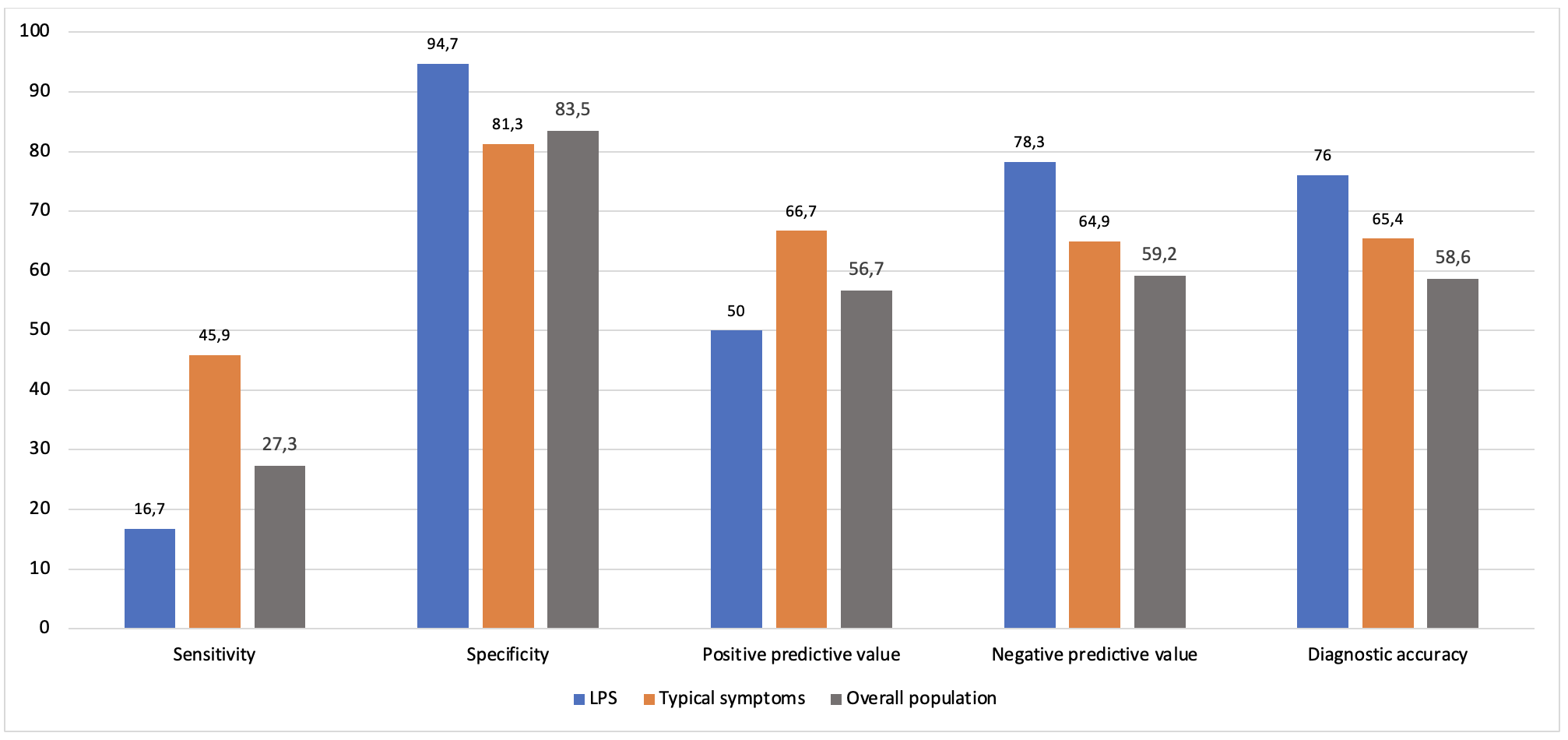

Supplement: Supplementary file 1 — Figure S1. Comparison of performance metrics of PPI response for the diagnosis of GERD in LPS versus typical symptoms. [file NMO-38-e70015-s001.tiff]
